# High-pressure crystal structure of *n*-hexyl­amine

**DOI:** 10.1107/S2053229625004504

**Published:** 2025-05-27

**Authors:** Bernadetta Kuleczka, Natalia Sacharczuk, Anna Olejniczak, Marcin Podsiadło

**Affiliations:** a Faculty of Chemistry, Adam Mickiewicz University, Uniwersytetu Poznańskiego 8, 61-614 Poznań, Poland; University of Strathclyde, United Kingdom

**Keywords:** hexyl­amine, crystal structure, N—H⋯N hy­dro­gen bonds, aliphatic amines, *in-situ* crystallization, high pressure, com­pressibility

## Abstract

A primary amine, *n*-hexyl­amine (**HA**, C_6_H_15_N), has been studied at high pressure by single-crystal X-ray diffraction. The structure of this com­pound has been determined, at ambient tem­per­a­ture, from the freezing pressure up to 1.40 GPa. **HA** at high pressure crystallizes in the space group *Pca*2_1_, which was already found at ambient pressure and low tem­per­a­ture. The com­pressibility of the N—H⋯N hy­dro­gen bonds has been com­pared with that of the shortest C—H⋯N and H⋯H inter­molecular distances, revealing that the H⋯H distances exhibit the highest degree of com­pressibility among them.

## Abstract 2

The high-pressure crystal structure of *n*-hexyl­amine remains isostructural with its low-tem­per­a­ture phase. The com­pressibility of the N—H⋯N hy­dro­gen bonds has been analyzed in relation to the shortest C—H⋯N and H⋯H inter­molecular distances.

## Introduction

N—H⋯N hy­dro­gen bonds often com­pete with weaker C—H⋯N hy­dro­gen bonds in crystals (Huang *et al.*, 2013 ▸; Leigh *et al.*, 2013 ▸; Podsiadło *et al.*, 2017 ▸; Sacharczuk *et al.*, 2023 ▸; Vega *et al.*, 2005 ▸). The role of these inter­actions, as the main cohesion forces in crystals, has been well documented for biomolecules, self-organizing materials, pharmaceuticals and mol­ecular switches (Desiraju & Steiner, 2001 ▸; Jeffrey & Saenger, 1994 ▸). The simplest aliphatic amines serve as model com­pounds for studying the nature of such inter­actions due to their mol­ecular com­position and the minimized effect of the mol­ecular shape on dense packing.
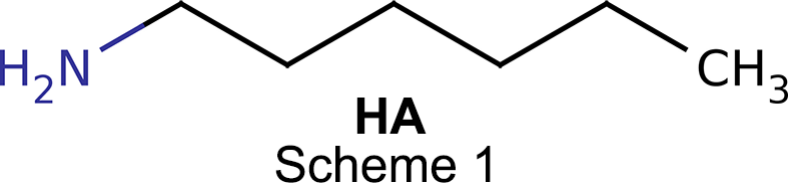


Structural studies of the simplest *n*-aliphatic amines have been per­formed at low tem­per­a­ture and ambient pressure for methyl­amine (Atoji & Lipscomb, 1953 ▸), as well as for the series from ethyl­amine to *n*-decyl­amine (Maloney *et al.*, 2014 ▸). These studies described the role of various types of inter­molecular inter­actions in mol­ecular association. N—H⋯N hy­dro­gen bonds have been identified as the main cohesion force for the early primary amines; however, the role of dispersive inter­actions between alkyl chains was also emphasized (for the later com­pounds, the dispersion inter­actions dominate over hy­dro­gen bonding). It was also found that dispersive inter­actions were particularly dominant in the regions between mol­ecular layers linked by N—H⋯N hy­dro­gen bonds (Maloney *et al.*, 2014 ▸).

**Figure 1:**
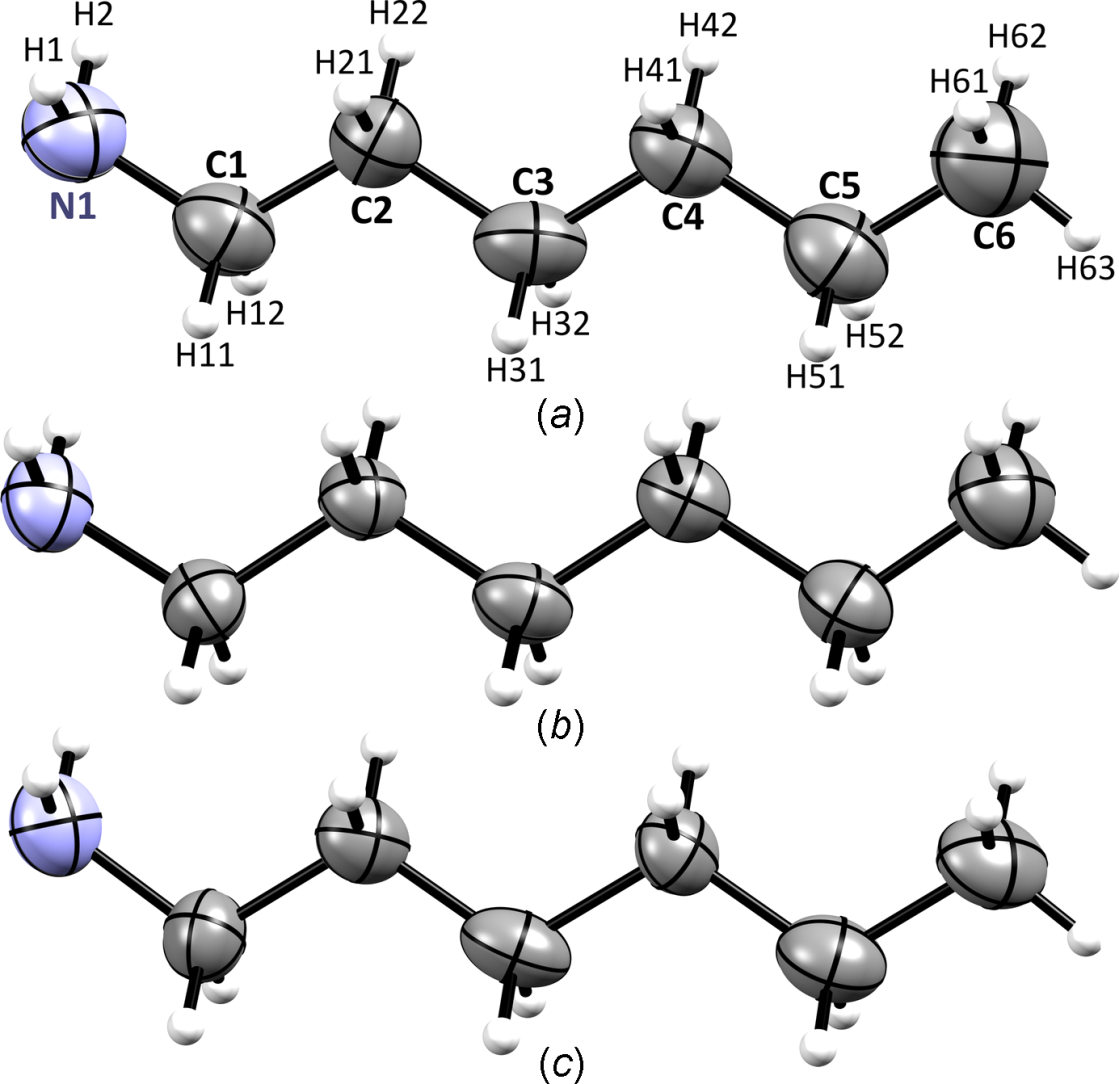
The mol­ecular structures of **HA** at (*a*) 0.50 GPa, (*b*) 0.65 GPa and (*c*) 1.40 GPa (all at 295 K), showing the atomic labelling scheme. Displacement ellipsoids are drawn at the 50% probability level.

This study of *n*-hexyl­amine (**HA**; Scheme 1 and Fig. 1 ▸) extends our previous research on the simplest primary amines under high pressure. Recently, we have investigated the series from methyl­amine to *n*-pentyl­amine (Podsiadło *et al.*, 2017 ▸; Sacharczuk *et al.*, 2023 ▸). Only ethyl­amine crystallizes in the same phase under high pressure (phase II) as found at low tem­per­a­ture. In contrast, seven new polymorphs, different from the low-tem­per­a­ture ones, have been identified at high pressure for the remaining amines, *i.e.* one for methyl­amine (Podsiadło *et al.*, 2017 ▸) and two each for propyl­amine, butyl­amine and pentyl­amine (Sacharczuk *et al.*, 2023 ▸). In all these polymorphs, mol­ecules inter­act through N—H⋯N hy­dro­gen bonds. However, at high pressure, the role of C—H⋯N hy­dro­gen bonds increases (Sacharczuk *et al.*, 2023 ▸).

In the present study, we have investigated **HA** at high pressure using single-crystal X-ray diffraction. The crystal structure has been determined previously at ambient pressure and low tem­per­a­ture only (Maloney *et al.*, 2014 ▸). We have obtained and investigated single crystals of **HA** in a diamond-anvil cell (DAC) in the range between the freezing pressure at ambient tem­per­a­ture of 0.33 GPa up to 1.40 GPa.

## Experimental

**Figure 2:**
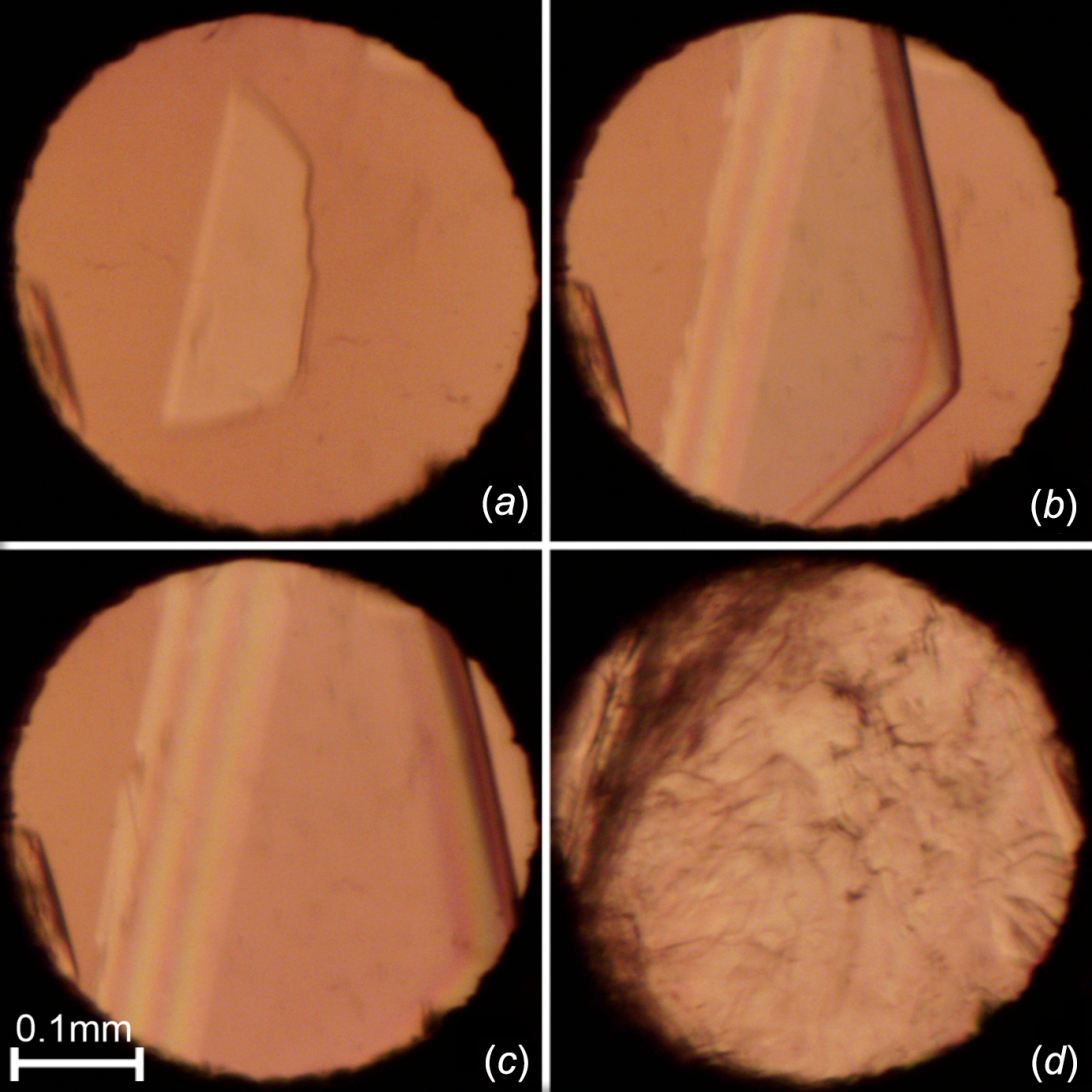
Stages of the **HA** single-crystal growth inside the DAC chamber (polarized-light mode): (*a*) one crystal seed at 295 K and 0.33 GPa, (*b*)/(*c*) the single-crystal growth with increasing pressure and a simultaneous decrease in the volume of the high-pressure chamber, and (*d*) the single crystal filling the DAC chamber at 295 K and 0.50 GPa. The ruby chip, for pressure calibration, is located by the left edge of the DAC.

*n*-Hexyl­amine, **HA** (99%), from Sigma–Aldrich was crystallized *in situ* in a modified Merrill–Basset diamond-anvil cell (DAC; Bassett, 2009 ▸). The DAC was equipped with a 0.3 mm thick steel gasket with a hole of diameter 0.3 mm. At 295 K, **HA** froze at 0.33 GPa, in the form of a polycrystalline mass, filling the whole volume of the DAC chamber. A single crystal of **HA** was obtained under isothermal conditions. The polycrystalline mass was melted, except for one crystallite, by decreasing the pressure slowly. Then, again slowly, the pressure was increased allowing a single crystal of **HA** to grow and eventually fill the entire volume of the chamber (Fig. 2 ▸). Afterwards, the pressure was increased by *ca* 0.2 GPa to achieve a stable single crystal required for the X-ray diffraction measurement. Several attempts were made to grow a single crystal under isochoric conditions at pressures above 0.50 GPa; however, all were unsuccessful. The growing single crystals were very sensitive to tem­per­a­ture changes, and each time additional crystallites occurred, even with slow cooling. For this reason, single crystals at pressures of 0.65 and 1.40 GPa were obtained at room tem­per­a­ture by increasing the pressure in the chamber after per­forming the X-ray measurements at 0.50 and 0.65 GPa. The pressure was calibrated by the ruby fluorescence method (Mao *et al.*, 1986 ▸; Piermarini *et al.*, 1975 ▸) using a Photon Control spectrometer with an accuracy of 0.02 GPa. The calibrations were per­formed before and after each X-ray diffraction experiment. The progress of the growth of the **HA** single crystal is shown in Fig. 2 ▸ and Fig. S1 in the supporting information.

Rigaku Xcalibur EOS and Xcalibur ATLAS dif­frac­tom­eters were used for the high-pressure studies. The DAC was centred by the gasket-shadow method (Budzianowski & Katrusiak, 2004 ▸).

The room-tem­per­a­ture com­pressibility measurement was per­formed up to *ca* 2 GPa in a piston-and-cylinder apparatus (Baranowski & Moroz, 1982 ▸; Dziubek & Katrusiak, 2014 ▸) with an initial volume of *ca* 8.5 cm^3^.

### Refinement

Crystal data, data collection and structure refinement details are summarized in Table 1 ▸. The H atoms of the methyl­ene and methyl groups were located based on the mol­ecular geometry, with the C—H distances equal to 0.97 or 0.96 Å and their *U*_iso_ factors constrained to 1.2 or 1.5 times the *U*_eq_ value of their carrier. The H atoms of the amine (–NH_2_) group were located based on the mol­ecular geometry, assuming N—H distances equal to 0.90 Å, and their *U*_iso_ factors were constrained to 1.2 times the *U*_eq_ value of their carrier. The crystal data and refinement details are summarized in Tables 1 ▸ and 2 ▸, and Table S1 in the supporting information.

## Results and discussion

**Figure 3:**
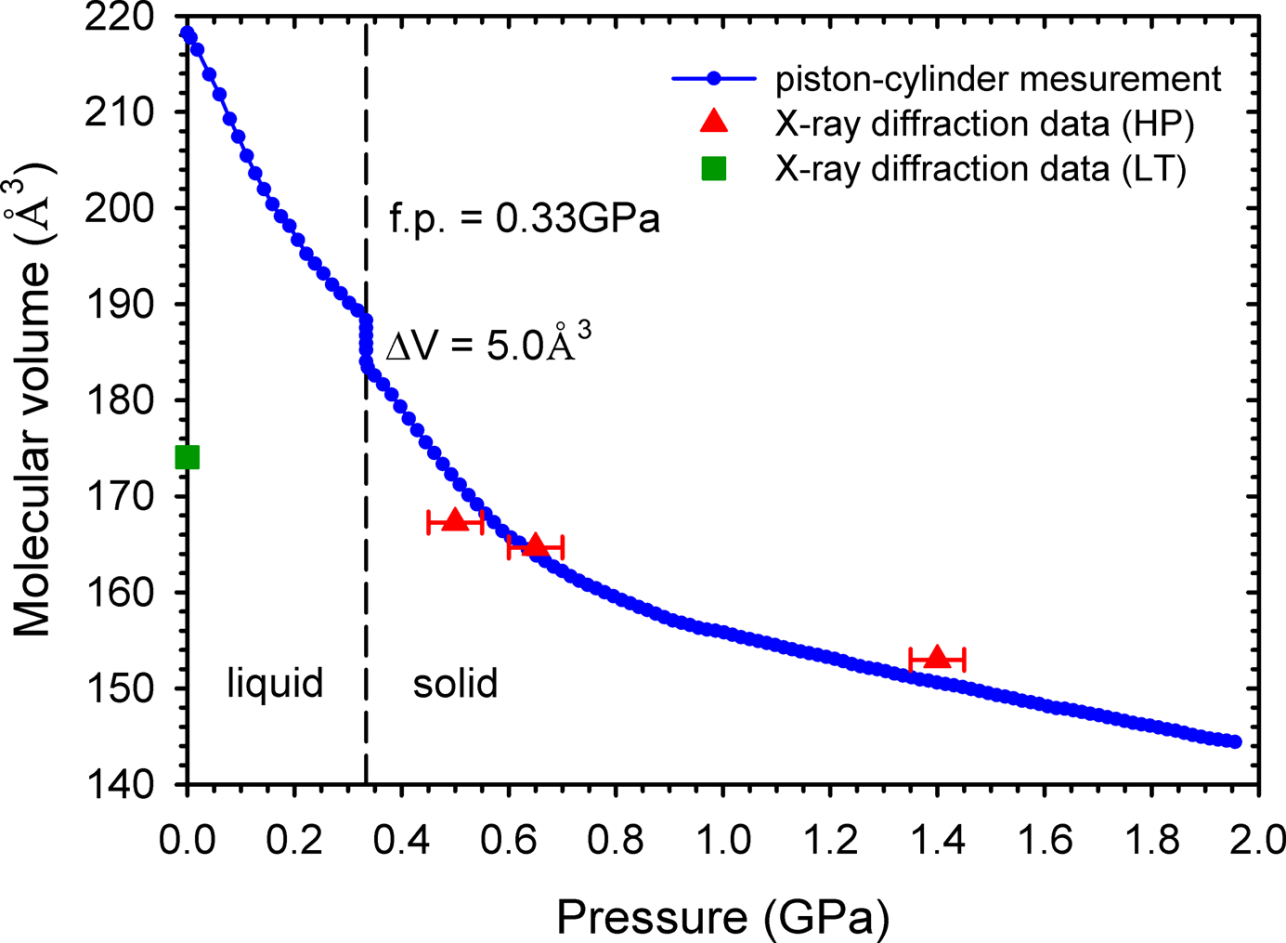
The mol­ecular volume of **HA** at room tem­per­a­ture as a function of pressure measured in the piston-and-cylinder press (blue circles). The volumes measured at high pressure (red triangles) and low tem­per­a­ture (green square; Maloney *et al.*, 2014 ▸) by single-crystal X-ray diffraction have been indicated. The freezing pressure (f.p.) of 0.33 GPa is marked with a vertical dashed line.

The single crystal of **HA** was obtained at the lowest possible pressure of 0.50 GPa (*ca* 0.2 GPa above the freezing pressure) to ensure the stability of the crystal during the X-ray diffraction data collection experiment. Two additional measurements were per­formed on this crystal com­pressed under isothermal conditions to 0.65 GPa and then to 1.40 GPa. The pressure of 1.40 GPa was the highest, at which the com­pressive stress on the crystal did not significantly affect the quality of the diffraction data. Above 1.40 GPa, the **HA** single crystal cracked due to mechanical stress. The crystals obtained under high pressure are denser than those crystallized at ambient pressure and low tem­per­a­ture (Table 2 ▸). All the unit-cell parameters of the investigated **HA** crystals decrease with increasing pressure, leading to the more dense structures. The mol­ecular volume of **HA** as a function of pressure has been plotted in Fig. 3 ▸.

The com­pression of the mol­ecular volume of **HA** measured at 295 K in a piston-and-cylinder press (Baranowski & Moroz, 1982 ▸; Dziubek & Katrusiak, 2014 ▸) changes abruptly by 2.6% on freezing the liquid at 0.33 GPa (Fig. 3 ▸). After freezing, the solid **HA** is initially strongly com­pressed until about 0.80 GPa; thereafter, the com­pression decreases monotonically, and at 1.96 GPa, the volume reaches about 65% of the liquid at 0.1 MPa and 80% of the solid at 0.33 GPa. The mol­ecular volume determined using the piston-and-cylinder press is consistent with that obtained by single-crystal X-ray diffraction (Fig. 3 ▸).

**Figure 4:**
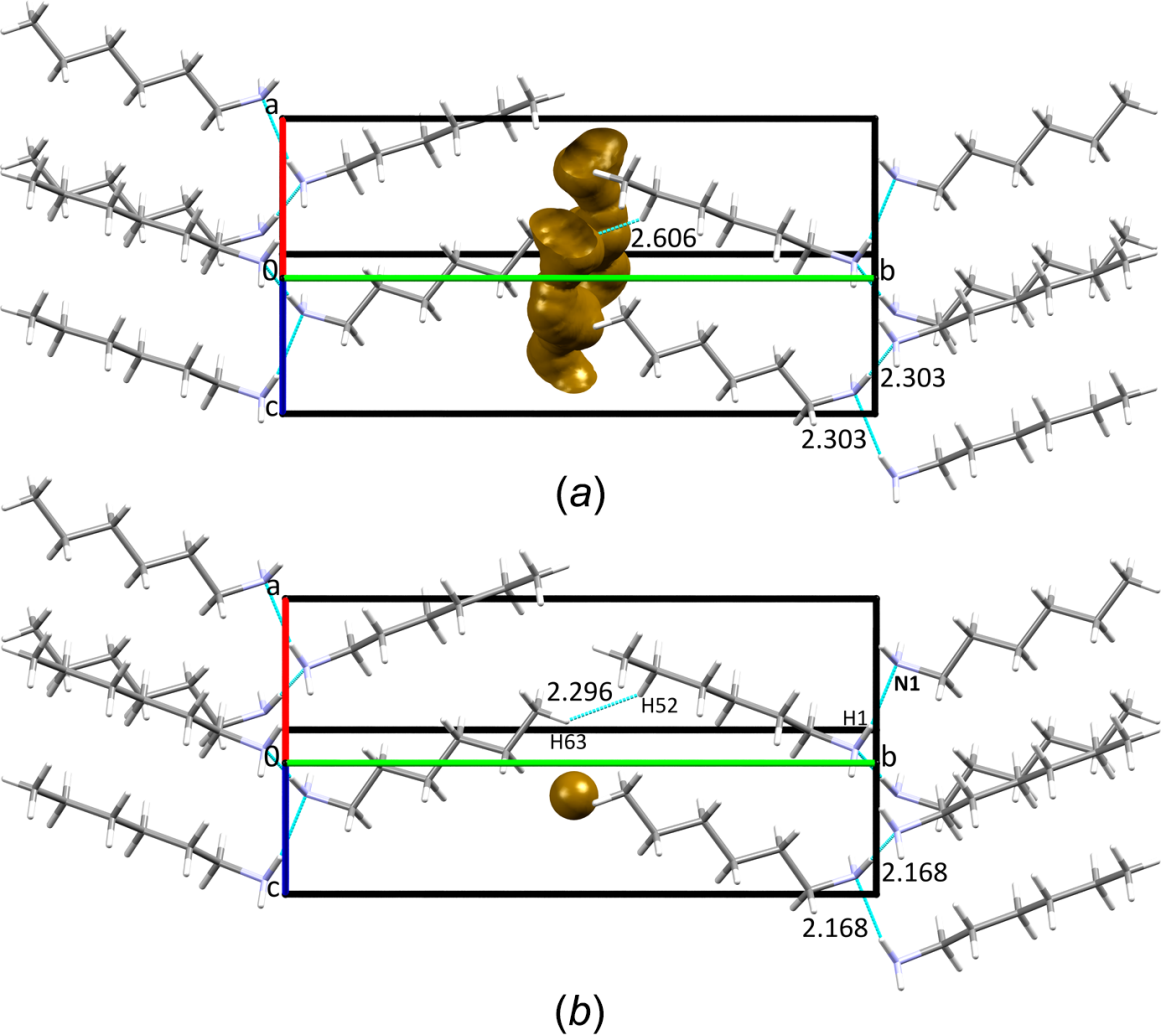
Structures of **HA** at low-tem­per­a­ture (Maloney *et al.*, 2014 ▸) and high-pressure conditions: (*a*) 0.1 MPa/150 K and (*b*) 1.40 GPa/295 K. Four hy­dro­gen bonds (N—H⋯N) and one short inter­molecular H⋯H distance at the end of the carbon chains are marked with dashed lines (distances are indicated). The inter­molecular space accessible to a probe with a radius of 0.7 Å and a grid spacing of 0.1 Å (Macrae *et al.*, 2020 ▸) is indicated in yellow.

**HA** at 0.1 MPa/150 K crystallizes in the noncentrosymmetric space group *Pca*2_1_, adopting a layered arrangement with infinite N—H⋯N hy­dro­gen-bonded chains running within the layers (Maloney *et al.*, 2014 ▸). These N—H⋯N hy­dro­gen-bonded chains can be described by the symbol 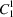
(2) according to the graph-set notation of hy­dro­gen bonds (Etter *et al.*, 1990 ▸). Within the chains, each N atom acts as a donor and an acceptor of an H atom. These chains are retained at high pressure when **HA** crystallizes in the same phase at 0.33 GPa/295 K (Fig. 4 ▸). This structure remains stable up to at least a pressure of 1.40 GPa.

N—H⋯N hy­dro­gen bonds play a major role in the cohesion force in **HA** crystals at ambient pressure/low tem­per­a­ture and within the investigated pressure range at room tem­per­a­ture. These inter­actions at ambient pressure/low tem­per­a­ture are characterized by inter­molecular H⋯N distances shorter by *ca* 0.45 Å (Maloney *et al.*, 2014 ▸) than the sum of the van der Waals radii of H and N atoms of 2.75 Å (Bondi, 1964 ▸) (Figs. 4 ▸ and 5 ▸). At 0.50 GPa, these distances are 2.229 (6) Å, and they are even shorter at 1.40 GPa, *i.e.* 2.168 (5) Å (Figs. 4 ▸ and 5 ▸, and Table 3 ▸).

**Figure 5:**
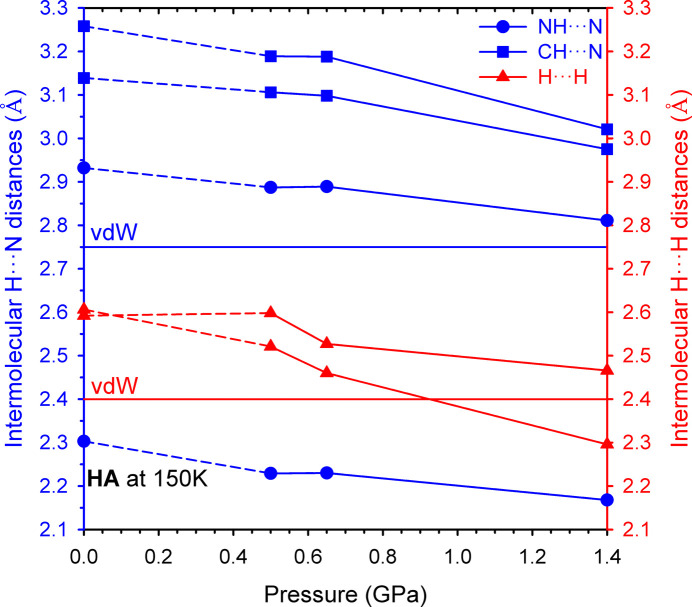
Inter­molecular H⋯N and H⋯H distances (Å) observed in **HA** structures plotted as a function of pressure. The two shortest distances for each type are presented: blue circles represent the N—H⋯N and blue squares depict the C—H⋯N hy­dro­gen–acceptor (H⋯*A*) distances, while red triangles represent inter­molecular H⋯H distances. Blue and red horizontal lines show the sum of the van der Waals radii of H and N of 2.75 Å, and of two H atoms of 2.40 Å (Bondi, 1964 ▸). The estimated standard deviations are smaller than the plotted symbols.

It is characteristic that the second shortest inter­molecular H⋯N distances from the N—H⋯N hy­dro­gen bonding are approximately 0.6 Å longer than the shortest. Within the investigated pressure range, these distances remain longer than the sum of the van der Waals radii (Fig. 5 ▸). Such a property was observed for the first time in the high-pressure structures of the simplest primary amines (Podsiadło *et al.*, 2017 ▸; Sacharczuk *et al.*, 2023 ▸). Furthermore, even in the **HA** structure at 1.40 GPa, no inter­molecular H⋯N distances from C—H⋯N hy­dro­gen bonds shorter than the sum of the van der Waals radii are observed. **HA** is therefore the first primary *n*-amine in the series from methyl- to hexyl­amine where, at high pressure, only single N—H⋯N hy­dro­gen-bonded chains are observed. The C—H⋯N hy­dro­gen bonds are not formed at all and no phase transition, that would allow mol­ecular rearrangement and the formation of C—H⋯N inter­actions, occurred (Podsiadło *et al.*, 2017 ▸; Sacharczuk *et al.*, 2023 ▸). In the series from methyl- to pentyl­amine, high pressure does not affect the main cohesive force (N—H⋯N hy­dro­gen bonds); however, inter­molecular C—H⋯N inter­actions are observed in the high-pressure polymorphs (Podsiadło *et al.*, 2017 ▸; Sacharczuk *et al.*, 2023 ▸).

In the **HA** crystals, only at a pressure of 1.40 GPa do the shortest inter­molecular H⋯H distances, at the end of the carbon chains, become shorter than the sum of the van der Waals radii of two H atoms of 2.4 Å (Bondi, 1964 ▸) (Figs. 4 ▸ and 5 ▸, and Fig. S2 in the supporting information). It is characteristic that, with increasing pressure, these shortest inter­molecular H⋯H distances are more com­pressible than the main N—H⋯N contacts (Figs. 4 ▸ and 5 ▸). This is related to the voids observed in the crystals between the ends of the carbon chains (Fig. 4 ▸).

In the series from methyl- to hexyl­amine, **HA** forms the least dense crystals in the structures determined just above their freezing pressure. The crystal density of methyl­amine determined at 3.65 GPa is 1.165 g cm^−3^ (Podsiadło *et al.*, 2017 ▸), ethyl­amine at 1.40 GPa is 1.046 g cm^−3^ (Sacharczuk *et al.*, 2023 ▸), propyl­amine at 2.25 GPa is 1.109 g cm^−3^ (Sa­char­czuk *et al.*, 2023 ▸), butyl­amine at 1.45 GPa is 1.059 g cm^−3^ (Sacharczuk *et al.*, 2023 ▸) and pentyl­amine at 1.05 GPa is 1.049 g cm^−3^ (Sacharczuk *et al.*, 2023 ▸). The crystal density of **HA**, determined within this study, at 0.50 GPa is 1.005 g cm^−3^. This correlates with the void volumes in the structures mentioned above, which, in the series from methyl- to hexyl­amine, are 0, 7.15, 7.86, 12.94, 13.65 and 38.45 Å^3^, respectively (the inter­molecular space accessible to a probe with a radius of 0.6 Å and a grid spacing of 0.1 Å; the parameters used in the calculations differ from those used in Fig. 4 ▸, ensuring distinguishable void volumes across the entire series; Macrae *et al.*, 2020 ▸).

## Conclusions

The high-pressure crystal structure of **HA** is isostructural with the phase determined at low tem­per­a­ture at 0.1 MPa (Maloney *et al.*, 2014 ▸). This represents the first example among the series of the simplest aliphatic amines where the crystal symmetry remains unchanged between low-tem­per­a­ture and high-pressure conditions. The space group symmetry of *Pca*2_1_ remains stable within the investigated pressure range. Similar to other aliphatic *n*-amines, the main cohesion force in the **HA** crystals involves the N—H⋯N hy­dro­gen-bonded chains. However, no additional inter­molecular distances shorter than the sum of the van der Waals radii are observed. This is unique within the high-pressure studies of the methyl- to pentyl­amine series, where high pressure enhances the role of inter­molecular C—H⋯N inter­actions. Only at 1.40 GPa does the first inter­molecular H⋯H distance become shorter than the sum of the van der Waals radii of two H atoms. The com­pressibility of this distance exceeds that of the inter­molecular N—H⋯N hydrogen bonds. This effect results from the voids surrounding the methyl groups at the ends of the carbon chains. The **HA** crystal obtained just above the freezing pressure is the least dense structure among the high-pressure structures determined in the methyl- to hexyl­amine series.

## Supplementary Material

Crystal structure: contains datablock(s) hexylamine_0_50GPa, hexylamine_0_65GPa, hexylamine_1_40GPa, global. DOI: 10.1107/S2053229625004504/vp3043sup1.cif

Structure factors: contains datablock(s) hexylamine_0_50GPa. DOI: 10.1107/S2053229625004504/vp3043hexylamine_0_50GPasup2.hkl

Supporting information file. DOI: 10.1107/S2053229625004504/vp3043hexylamine_0_50GPasup5.cml

Structure factors: contains datablock(s) hexylamine_0_65GPa. DOI: 10.1107/S2053229625004504/vp3043hexylamine_0_65GPasup3.hkl

Structure factors: contains datablock(s) hexylamine_1_40GPa. DOI: 10.1107/S2053229625004504/vp3043hexylamine_1_40GPasup4.hkl

Supporting information file. DOI: 10.1107/S2053229625004504/vp3043hexylamine_0_50GPasup5.cml

Additional figures and table. DOI: 10.1107/S2053229625004504/vp3043sup6.pdf

CCDC references: 2434096, 2434097, 2434098

## Figures and Tables

**Table 1:** Experimental details For all determinations: C_6_H_15_N, *M*_r_ = 101.19, orthorhombic, *Pca*2_1_, *Z* = 4. Experiments were carried out at 295 K with Mo *K*α radiation. Absorption was corrected for by multi-scan methods (*CrysAlis PRO*; Rigaku OD, 2022 ▸). Refinement was on 64 parameters with 1 restraint. H-atom parameters were constrained.

	**HA** at 0.50 GPa	**HA** at 0.65 GPa	**HA** at 1.40 GPa
Crystal data			
*a*, *b*, *c* (Å)	6.9050 (9), 17.549 (7), 5.5212 (13)	6.8591 (9), 17.494 (5), 5.4892 (4)	6.7241 (19), 17.052 (13), 5.3367 (7)
*V* (Å^3^)	669.1 (3)	658.7 (2)	611.9 (5)
μ (mm^−1^)	0.06	0.06	0.06
Crystal size (mm)	0.28 × 0.28 × 0.27	0.27 × 0.27 × 0.26	0.26 × 0.26 × 0.23

Data collection			
Diffractometer	Rigaku Xcalibur Eos	Rigaku Xcalibur Atlas	Rigaku Xcalibur Atlas
*T*_min_, *T*_max_	0.573, 1.000	0.600, 1.000	0.447, 1.000
No. of measured, independent and observed [*I* > 2σ(*I*)] reflections	4308, 832, 373	5447, 1085, 527	4114, 852, 401
*R*_int_	0.071	0.063	0.068
(sin θ/λ)_max_ (Å^−1^)	0.663	0.732	0.713

Refinement			
*R*[*F*^2^ > 2σ(*F*^2^)], *wR*(*F*^2^), *S*	0.054, 0.118, 1.04	0.047, 0.131, 0.98	0.061, 0.194, 1.04
No. of reflections	832	1085	852
Δρ_max_, Δρ_min_ (e Å^−3^)	0.09, −0.09	0.10, −0.10	0.17, −0.20
Absolute structure	Flack *x* determined using 128 quotients [(*I*^+^) − (*I*^−^)]/[(*I*^+^) + (*I*^−^)] (Parsons *et al.*, 2013 ▸)	Flack *x* determined using 181 quotients [(*I*^+^) − (*I*^−^)]/[(*I*^+^) + (*I*^−^)] (Parsons *et al.*, 2013 ▸)	Flack *x* determined using 130 quotients [(*I*^+^) − (*I*^−^)]/[(*I*^+^) + (*I*^−^)] (Parsons *et al.*, 2013 ▸)
Absolute structure parameter	−10.0 (10)	0.7 (10)	3.1 (10)

Computer programs: *CrysAlis PRO* (Rigaku OD, 2022 ▸), *SHELXT2018* (Sheldrick, 2015*a* ▸), *SHELXL2018* (Sheldrick, 2015*b* ▸), *Mercury* (Macrae *et al.*, 2020 ▸) and *OLEX2* (Dolomanov *et al.*, 2009 ▸).

**Table 2:** Selected crystal data of **HA** at 0.1 MPa/150 K and 0.50, 0.65 and 1.40 GPa (all at 295 K)

	C_6_H_15_N^*a*^	C_6_H_15_N^*b*^	C_6_H_15_N^*b*^	C_6_H_15_N^*b*^
*p* (GPa)	0.0001	0.50 (2)	0.65 (2)	1.40 (2)
*T* (K)	150	295 (2)	295 (2)	295 (2)
Crystal system	Ortho­rhom­bic	Ortho­rhom­bic	Ortho­rhom­bic	Ortho­rhom­bic
Space group	*Pca*2_1_	*Pca*2_1_	*Pca*2_1_	*Pca*2_1_
*a* (Å)	6.9725 (3)	6.9050 (9)	6.8591 (9)	6.7241 (19)
*b* (Å)	17.7977 (6)	17.549 (7)	17.494 (5)	17.052 (13)
*c* (Å)	5.6105 (2)	5.5212 (13)	5.4892 (4)	5.3367 (7)
*V* (Å^3^)	696.23 (5)	669.1 (3)	658.7 (2)	611.9 (5)
*Z*, *Z*′	4, 1	4, 1	4, 1	4, 1
*D_*x*_* (g cm^−3^)	0.965	1.005	1.020	1.098
*R*_1_ [*F*^2^ > 2σ(*F*^2^)]	0.0418	0.0544	0.0472	0.0612
*R*_1_ (all data)	-	0.1726	0.1434	0.1624

References: (*a*) Maloney *et al.* (2014 ▸); (*b*) this work.

**Table 3:** Hydrogen-bond geometry (Å, °)

N1—H1⋯N1^i^	0.50 GPa	0.65 GPa	1.40 GPa
N1—H1	0.900 (7)	0.900 (4)	0.900 (7)
H1⋯N1^i^	2.229 (6)	2.230 (3)	2.168 (5)
N1⋯N1^i^	3.116 (7)	3.115 (4)	3.050 (8)
N1—H1⋯N1^i^	168.2 (2)	167.44 (13)	166.4 (2)

Symmetry code: (i) −*x* + 1, −*y*, *z* − 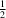.
