# Supplementary material for: High-pressure crystal structure of n-hexyl­amine
Source: Acta Crystallogr C Struct Chem. 2025 May 27;81(Pt 6):346–50. doi: 10.1107/S2053229625004504 (PMC12138252; doi:10.1107/S2053229625004504)
Supplement: Supplementary file 6 [file c-81-00346-sup6.pdf]

STRUCTURAL CHEMISTRY

Volume 81 (2025)

Supporting information for article:

High-pressure crystal structure of *n*-hexylamine

Bernadetta Kuleczka, Natalia Sacharczuk, Anna Olejniczak and Marcin Podsiadło

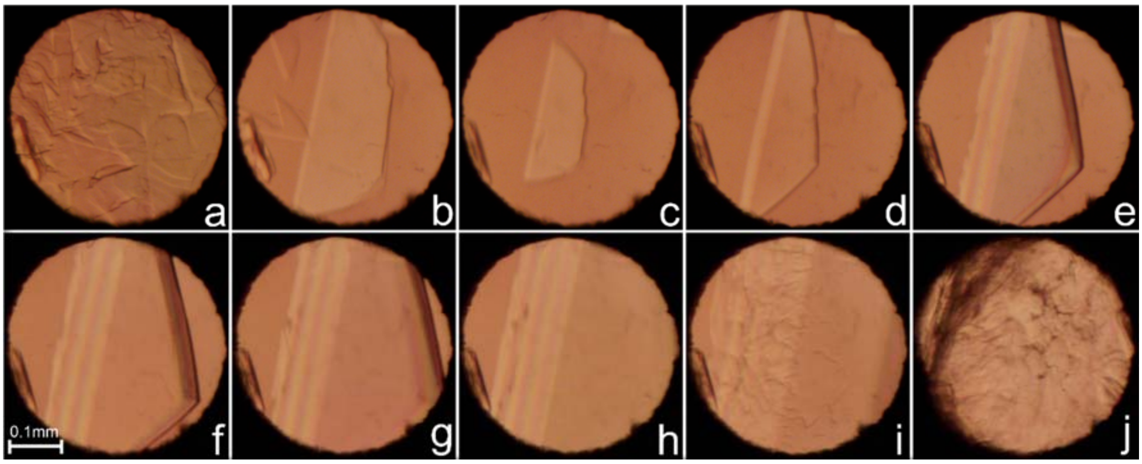

Figure S1 Stages of the *n*-hexylamine (**HA**) single-crystal growth inside the DAC chamber (polarized-light mode): (a) polycrystalline mass grown isothermally at 295 K and 0.37 GPa; (b) three single crystals–liquid equilibrium at 295 K and 0.33 GPa; (c) one crystal seed at 295 K and 0.33 GPa; (d-i) the single crystal growth during increasing pressure and simultaneous decrease in the volume of high-pressure chamber and (j) the single crystal filling the DAC chamber at 295 K and 0.50 GPa. The ruby chip, for pressure calibration, is located in the left part of the DAC.

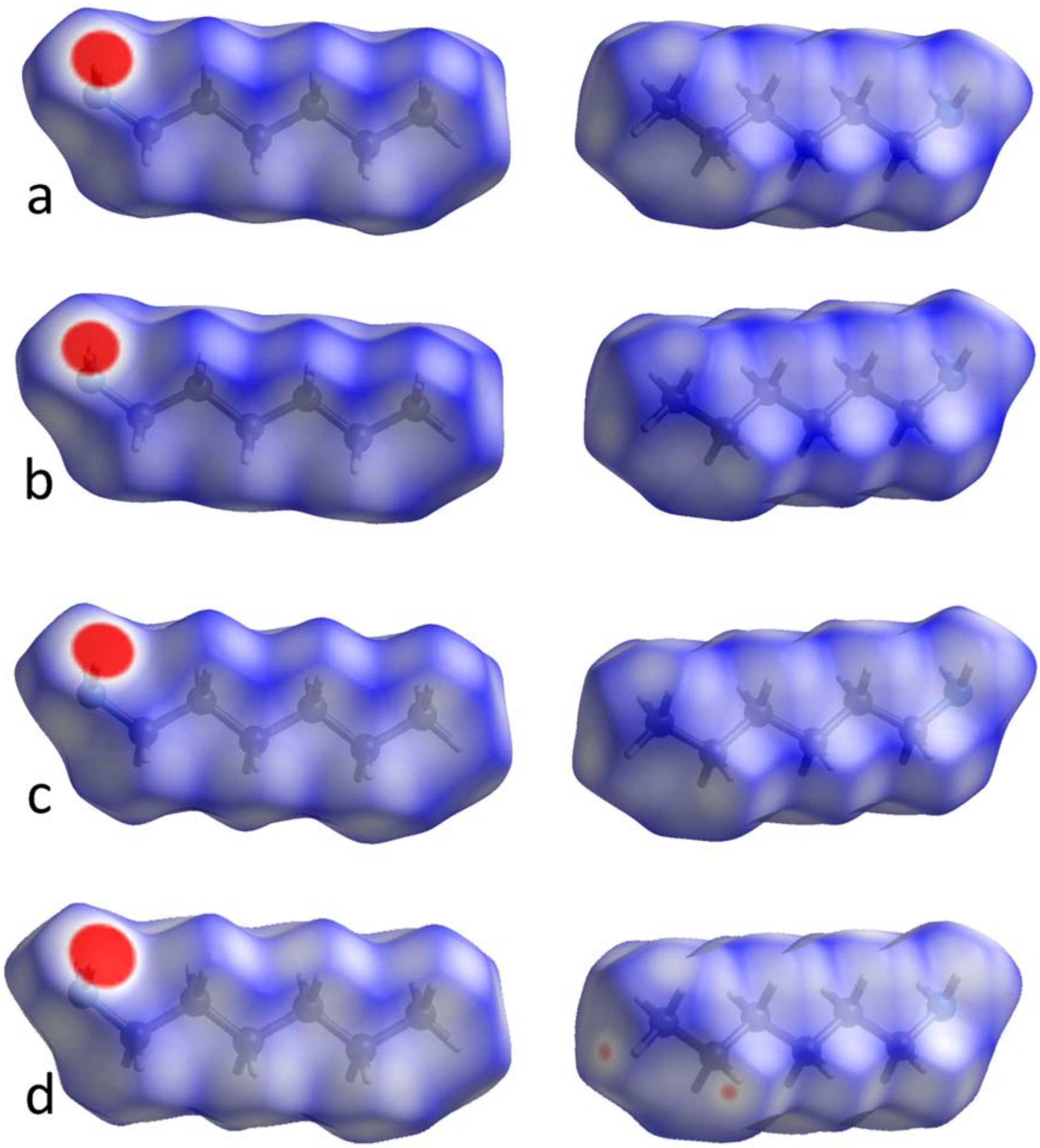

Figure S2 Hirshfeld surfaces (Spackman, P. R.; Turner, M. J.; McKinnon, J. J.; Wolff, S. K.; Grimwood, D. J.; Jayatilaka, D.; Spackman, M. A. *CrystalExplorer21*: a program for Hirshfeld surface analysis, visualization and quantitative analysis of molecular crystals, *J. Appl. Cryst*., **2021**, *54*, 1006-1011) mapped with d_{norm} (from −0.1 to 1.0) of the **HA** molecules at: 0.1 MPa/150 K (Maloney, A. G. P.; Wood, P. A.; Parsons, S. Competition between hydrogen bonding and dispersion interactions in the crystal structures of the primary amines, *CrystEngComm*. **2014**, *16*, 3867-3882) (a), 0.50 GPa/295 K (b), 0.65 GPa/295 K (c), and 1.40 GPa/295 K (d). The white, red and blue colours indicate contacts that are equal, shorter and longer, respectively, than the sums of the van der Waals radii of respective atoms (Bondi, A. van der Waals Volumes and Radii, *J. Phys. Chem.* **1964**, *68*, 441-451).

Table S1 Crystal data and details of the refinements of **HA** at 0.50, 0.65 and 1.40 GPa (all at 295 K).

	**$C_6H_{15}N$**	**$C_6H_{15}N$**	**$C_6H_{15}N$**
Pressure (GPa)	0.50(2)	0.65(2)	1.40(2)
Formula weight	101.19	101.19	101.19
Crystal colour	colourless	colourless	colourless
Crystal size (mm)	0.28x0.28x0.27	0.27x0.27x0.26	0.26x0.26x0.23
Crystal system	orthorhombic	orthorhombic	orthorhombic
Space group	$Pca2_1$	$Pca2_1$	$Pca2_1$
Unit cell dimensions			
a (Å)	6.9050(9)	6.8591(9)	6.7241(19)
b (Å)	17.549(7)	17.494(5)	17.052(13)
c (Å)	5.5212(13)	5.4892(4)	5.3367(7)
Volume (Å^3)	669.1(3)	658.7(2)	611.9(5)
Z, *Z'*	4, 1	4, 1	4, 1
D_x (g·cm^{-3})	1.005	1.020	1.098
Wavelength MoKα, λ (Å)	0.71073	0.71073	0.71073
Absorption coefficient (mm^{-1})	0.059	0.059	0.064
F(000) (e)	232	232	232
2Θ max (°)	56.27	62.71	60.87
Min./Max. indices *h*, *k*, *l*	-9/9, -16/16, -6/6	-8/8, -15/15, -7/7	-8/8, -14/13, -7/7
Reflections collected/unique	4308/832	5447/1085	4114/852
R_{int} (all data)	0.0707	0.0630	0.0680
Observed reflections ($I>2\sigma(I)$)	373	527	401
Data/restraints/parameters	832/1/64	1085/1/64	852/1/64
Goodness of fit on F^2	1.039	0.977	1.038
Final R_1 indices ($I>2\sigma(I)$)	0.0544	0.0472	0.0612
R_1/wR_2 indices (all data)	0.1726/0.1180	0.1434/0.1313	0.1624/0.1943
$\Delta\sigma_{max}$, $\Delta\sigma_{min}$ (e·Å^{-3})	0.09/-0.09	0.10/-0.10	0.17/-0.19
Weighting scheme: x; y^a	0.0376; 0	0.0573; 0	0.0896; 0

$^a w=1/(\sigma^2(Fo^2)+x^2P^2+yP)$, where $P=(\mathrm{Max}(Fo^2,0)+2Fc^2)/3$
